# Exploring nurses’ experiences of value congruence and the perceived relationship with wellbeing and patient care and safety: a qualitative study

**DOI:** 10.1177/1744987120976172

**Published:** 2021-01-10

**Authors:** Alice Dunning, Gemma Louch, Angela Grange, Karen Spilsbury, Judith Johnson

**Affiliations:** PhD Student, School of Psychology, 4468 University of Leeds, UK; 170791 Bradford Institute for Health Research, UK; Senior Research Fellow, 170791 Bradford Institute for Health Research, UK; Head of Nursing, Research and Innovation, 170791 Bradford Institute for Health Research, UK; Professor, School of Healthcare, 4468 University of Leeds, UK; Lecturer, School of Psychology, 4468 University of Leeds, UK; 170791 Bradford Institute for Health Research, UK

**Keywords:** burnout, interview, nurses, patient care, patient safety, qualitative, values, wellbeing

## Abstract

**Background:**

Values are of high importance to the nursing profession. Value congruence is the extent to which an individual’s values align with the values of their organisation. Value congruence has important implications for job satisfaction.

**Aim:**

This study explored nurse values, value congruence and potential implications for individual nurses and organisations in terms of wellbeing and patient care and safety.

**Method:**

Fifteen nurses who worked in acute hospital settings within the UK participated in semi-structured telephone interviews. Thematic analysis was utilised to analyse the data.

**Results:**

Four themes were identified: organisational values incongruent with the work environment; personal and professional value alignment; nurse and supervisor values in conflict; nurses’ values at odds with the work environment. Perceived value incongruence was related to poorer wellbeing, increased burnout and poorer perceived patient care and safety. The barriers identified for nurses being able to work in line with their values are described.

**Conclusions:**

Value congruence is important for nurse wellbeing and patient care and safety. Improving the alignment between the values that organisations state they hold, and the values implied by the work environment may help improve patient care and safety and support nurses in practice.

## Introduction

Values are recognised as important within nursing (Rassin, 2008), and recent policy initiatives to recruit nursing staff based on their values underlines the centrality of values for the profession (NHS England, 2012). All Registered Nurses (RNs) possess values that will influence their attitudes, behaviours and emotions. Being aware of values that motivate RNs supports them in practice. RNs without self-awareness of their motivating values may struggle with their professional role, whereas RNs with an understanding of their values often achieve personal satisfaction (Altun, 2002). Studies have revealed a relationship between RN values and concepts of wellbeing such as levels of burnout (Saito et al., 2018) and job satisfaction, and performance outcomes (Atefi et al., 2014). Importantly, these studies explored either personal values (Atefi et al., 2014) or professional values (Ravari et al., 2013) and found both sets of values to influence RN job satisfaction and performance.

There is a growing awareness that healthcare staff wellbeing and work-related wellbeing concepts such as burnout may have implications for patient safety. Therefore, it is possible that associations between RN values and wellbeing concepts may also be relevant in terms of patient care and safety. For RNs who work in acute hospital settings, depressive symptoms have been found to be directly associated with poorer perceptions of patient safety at an individual and organisational level (Johnson et al., 2017). Furthermore, chronic stress has been negatively associated with perceptions of safety and the ability to act as a safe practitioner (Louch et al., 2017). These findings are concerning given that RNs perform a key role in ensuring patient safety as they monitor and co-ordinate patient care (Kirwin et al., 2013) and therefore have opportunities to reduce adverse events and prevent errors before they occur. RN staffing levels are a key issue in patient safety. A crucial review and discussion paper identified a positive relationship between RN staffing levels and patient outcomes including: lower death rates, reduced incidents of falls, shorter hospital stays and less missed care opportunities in acute hospital care settings (Griffiths et al., 2016). The relationship between RN staffing levels and patient outcomes is of particular concern in the UK due to the current shortfall, with more than 10% of nursing posts vacant (Buchan et al., 2019). Furthermore, research highlights that inadequate staffing impacts not only patient care and safety, but also negatively affects RN wellbeing (Sizmur and Raleigh, 2018).

Whilst professional and personal values have been studied simultaneously (Rassin, 2008), few studies have explored the relationship between these sets of values, and their association with organisational values. This is important as the relationships between wellbeing, burnout and patient care and safety are likely to be influenced by value congruence: the alignment of an individual employee’s values with those of the organisation in which they work (Verplanken, 2004). Value incongruence has been related to significant negative outcomes for RNs including low job satisfaction (Kaya et al., 2020), higher burnout (Leiter et al., 2009), greater intention to leave, decreased patient satisfaction (Gates and Mark, 2012) and increased staff turnover (Shao et al., 2018). One study found a significant inverse correlation between value congruence, job satisfaction and quality of patient care (Kramer and Hafner, 1989). However, there have been some mixed findings in this area, with one study concluding that value congruence was not related to job satisfaction (Kalliath et al., 1999), which underlines the need for further research to explore these relationships.

Studies to date highlight the importance of values for RNs and suggest potential relationships between value congruence, wellbeing and patient care and safety. However, these studies have predominantly used quantitative methods and there has been no study to date that explores RNs perceptions of these potential relationships.

### Aims

- To explore perceptions of values and value congruence with RNs employed in acute hospital settings.
- To understand how values and value congruence are perceived to be related to RN wellbeing, patient care and safety.

## Method

The study adopted an exploratory qualitative approach (Sandelowski, 2000). An essentialist approach was considered most appropriate as it reported the reality of the participant’s experiences and related meaning (Braun and Clarke, 2006). To guide and enhance the transparency of study reporting, the COREQ checklist was applied by one author (AD) (Tong et al., 2007).

### Design

Semi-structured telephone interviews were conducted with RNs working in acute hospital settings. Telephone interviews are a popular method with healthcare staff due to the flexibility of time and place they offer participants who work shifts (Carr and Worth, 2001). Research suggests that there is little difference in the responses yielded between telephone and face-to-face interviews (Carr and Worth, 2001). The interviews lasted an average of 30 minutes.

The interview schedule was informed by relevant literature (Altun, 2002; Atefi et al., 2014; Gates and Mark, 2012; Rassin, 2008; Verplanken, 2004) with several areas of focus including important values for nurses, value congruence and the implications of value congruence. The semi-structured interview style and schedule allowed for flexibility within the interview, which enabled the pursuit of issues raised by the study participants. Pilot interviews, conducted with research and community nurses (*n* = 2), helped refine the topic guide. The interview schedule followed an iterative approach in which earlier interviews with participants influenced subsequent interviews.

### Participant selection and recruitment

Any UK-based RN working in an acute hospital setting was eligible to participate. Nurses responded to advertisements on social media platforms (Facebook and Twitter) to register their interest in participating. Social media allowed for the recruitment of RNs from multiple organisations of different sizes from varied geographic locations with a broad range of experiences. This was to ensure the interviews included RNs’ perceptions from different organisational cultures. The study assumed a hybrid sampling method (Barber, 2001), using a combination of opportunity and purposive sampling. Opportunistic sampling was utilised in the first instance, an approach often used to recruit nurses (Barnfield et al., 2018). Following this, purposive sampling was embraced, which allowed a level of control over the cases sampled (Barber, 2001). The recruitment strategy followed an iterative approach, whereby the researcher (AD) engaged in preliminary analysis which shaped the subsequent sampled cases (Cohen and Crabtree, 2006). Subgroups not represented within the sample were subsequently targeted, for example different NHS bands and specialities.

Participants were recruited between May and November 2018. There were 26 responses to the advertisements, and 15 nurses completed the interviews. The nurses who initially expressed an interest in participating but did not complete an interview were not required to provide a reason for non-participation. However, those who did provide a reason for not being able to complete an interview cited differing work patterns or busy schedules. The participants were based across nine different hospitals which varied in geographical location and size, in England and Scotland. The majority of participants were female (93.3%), White British (93.3%) and Band 5 (66.66%; see Table 1).

**Table 1.** Participant characteristics within the sample.

Characteristic	Frequency (*n* (%))
Gender	
Male	1 (6.66)
Female	14 (93.3)
Ethnicity	
White British	14 (93.3)
Mixed other	1 (6.66)
Age	
21–24	6 (40)
25–34	3 (20)
35–44	3 (20)
45–54	1 (6.66)
55–64	2 (13.34)
NHS pay band (nurses.co.uk, 2020)	
5 (first level of RNs, will include newly registered nurses)	10 (66.66)
6 (more experienced nurses)	1 (6.66)
7 (advanced nurse or nurse practitioner)	3 (20)
8 (Matron or Senior Nurse)	1 (6.66)
Speciality	
Palliative care	1 (6.66)
Cardiac	1 (6.66)
Acute/general diseases	1 (6.66)
Infectious diseases	1 (6.66)
Renal medicine	1 (6.66)
Paediatrics	3 (20)
Acute cardiology	1 (6.66)
Critical care	1 (6.66)
General surgery	1 (6.66)
No speciality	2 (13.33)

Recruitment was ongoing until data saturation had been reached. Data saturation (Saunders et al., 2018) was considered to be achieved when new interview participants were not expressing new insights, thus leading to informational redundancy. One researcher (AD) listened to audio recordings of completed interviews in order to establish when informational redundancy was being reached.

### Data analysis

The interviews were audio-recorded and transcribed verbatim. Reflexive thematic analysis was used: this offers a flexible approach for data analysis to provide a rich and detailed account (Braun and Clarke, 2006). This was appropriate for understanding RNs’ values and their experiences of value congruence and its impact on wellbeing and patient care and safety. Data analysis involved six steps (Braun and Clarke, 2006). In the first step, familiarisation with the data, occurred through listening to the audio recordings, reading and re-reading transcripts with initial observations being noted. In the next stage, all transcripts were read and coded by one author (AD). Additionally, two authors independently coded a third of the transcripts (GL and JJ). After discussion and consensus between three authors (AD, GL and JJ) initial codes were generated and applied to the full dataset. In stage three, codes were gathered into potential themes. In the fourth stage, the themes were reviewed in relation to the coded extracts and the entire data set. In stage five, with further analysis, these themes were refined to generate clear definitions and names. Finally, meaningful extracts were identified to represent the themes. Throughout the analysis stages, one author (AD) simultaneously charted the data by creating tables with initial codes, pulling data from further transcripts into this and then visually grouping these to form the final themes.

## Results

Four key themes were identified, which described the different aspects of value congruence experienced by RNs: organisational values incongruent with the work environment; personal and professional value alignment; nurse and supervisor values in conflict; and nurses’ values at odds with the work environment.

### Organisational values incongruent with the work environment

There was incongruence described by most RNs across all bands, between organisational values and the work environments created in practice. The values that the RNs described as reported by their organisations included honesty, compassion, care, respect and being patient centred. However, the RNs perceived that organisations aimed to meet policy driven targets, and that managing limited resources and funding had become the most valued aspects for the organisations. Service pressures created barriers (e.g. staffing levels) which were perceived as preventing the nurses from being able to work in line with their values, eroding value congruence between RNs and their organisation. This incongruence was described by RNs from all bands (5–8), however the extent to which RNs viewed these pressures within the wider context varied. Band 5 RNs described this incongruence as being created at the organisation level, whereas higher banded RNs (i.e. 7, 8) described the incongruence as emerging due to external pressures from national policies or directives. Regardless of origin, this incongruence impacted on RNs’ perceived levels of wellbeing and feelings of wanting to leave the profession, across all bands. It also led to RNs feeling disenfranchised: RNs described their organisations as either using values as ‘buzz words’ or trying to enforce these values without creating an environment where it was possible for nurses to enact these to promote patient care and safety. The result was that this incongruence created tensions between the RNs and their organisations:

If I’m being totally honest I feel like they just tap these words out to like make them look good, but they don’t create an environment in order to fulfil them. So they say this is what we are striving for and this is what we are doing, but at the end of the day all it comes down to is money in the budget and that’s the most important thing to them like the managers and stuff. Like if I say, ‘well you know we want more staff so I can give person-centred care’, they would just say ‘well you should be giving that anyway’ erm and they say ‘we'll we have to look at the budget’ and that’s all they look at, is the numbers [*sic*]. (P6 Female, Band 5)

Nurses hate tick boxes they are just meaningless but governments love them but that’s not what patient care is all about […] I feel as though hands-on patient care is getting compromised by some of these things [*sic*]. (P15 Female, Band 7)

I think that hospitals that are under extreme pressure at the minute and I think that sometimes causes the conflict between your values and you know your actions. [*sic*] (P4 Male, Band 8)

### Personal and professional value alignment

There was a clear perceived relationship between personal and professional values. RNs described these as being inseparable and that both were integral to their role. RNs highlighted a specific value set required to be, and perform as, a nurse. The values most frequently described as important were related to both personal and professional life, for example, compassion and respect for others, integral to promoting patient experiences of care and safety. The interplay between personal and professional values with those of the workplace was considered important for RN wellbeing. Any value congruence or incongruence experienced by RNs within the workplace applied to their personal values, as well as professional:

I think what you bring to nursing is what you value, you know you can’t draw a line in the sand between them both you know what is important to you outside of work is always going to transfer to what you do inside of work and vice versa. [*sic*] (P12 Female, Band 7)

I feel like to be considered a nurse you have to have values that match up with the professional remit otherwise there are going to be issues. [*sic*] (P14 Female, Band 5)

### Nurse and supervisor values in conflict

Some RNs described a lack of congruence between their own values and the values held by their supervisors. There was a difference between the positions of supervision identified between the different bands of RNs. Band 5 RNs mainly discussed their immediate leaders on their ward or unit (e.g. matron, lead nurse); whereas RNs in higher bands (Band 7) more often referred to the management tier of their organisation or of the NHS. RNs described some of their supervisors as holding a different set of values to themselves, which could lead to supervisors asking or expecting them to behave in a way that was not in line with their own values. For example, Band 5 RNs reported the importance (to them) of providing good quality and safe patient care, whilst they perceived their supervisor’s values were related more to efficiency or numbers. Many RNs described their supervisors as prioritising the saving of money, conducting audits or managing the flow of patients through the hospital. This perceived values conflict was viewed as having an impact on patient safety, as nurses described being asked to act in a way that led to some potentially unsafe behaviours. Furthermore, the perception of a different value set among senior nurses also impacted upon levels of wellbeing or burnout. The quality of patient care that nurses felt they could provide was considered to be closely linked with their wellbeing. Nurses who described experiencing a conflict in values with those of their supervisors, felt they were unable to provide the care they wanted to provide, and perceived this as being linked to poorer wellbeing:

The Matron [the head of a nursing team; they carry out RN duties, but also look after a team of staff (nurses.co.uk, 2020)] asked me to move this patient that was close to dying on to the corridor. I was just like that is ridiculous obviously that goes against all your values but then so stuff like that and when they have patients on corridors and stuff like that. That was really hard to see patients on corridors, but not in bed areas. That goes against your values, like imagine coming to see your relative in hospital and they’re on the corridor. [*sic*] (P8 Female, Band 5)

When it goes higher up it will always come down to money and that’s where you will kind of lose your sense of values and because it’s not about the care anymore it’s about the business when it gets to the top of the NHS I think that upset me quite a lot because I didn’t feel like it was something that I could control. [*sic*] (P 13, Female, Band 7)

### Nurse values at odds with work environment

RNs discussed that care they were able to deliver within the current system was not in line with their own values. They valued providing high-quality, compassionate patient-centred care. However, service pressures and the demand on staff created a work environment which was incongruent with RN values, as these staff were no longer able to dedicate time to provide patient care. Building upon the previous theme, the discord within the work environment described by RNs was exacerbated by incongruence with supervisors. However, the incongruence within the work environment was a culmination of many factors. The challenges described within the work environment influenced the quality and safety of care that RNs perceived they could deliver. This inability to deliver safe and good quality patient care impacted on RNs’ impression of their wellbeing, and feelings of wanting to leave the profession:

The ability to be able to deliver care that is in line with your values is a massive influence on job satisfaction and being happy at work. So yeah absolutely, it is important for those elements of staff wellbeing that they are able to deliver nursing… that they are able to feel that they are delivering nursing that’s important to them. [*sic*] (P 12 Female, Band 7)

Sometimes that can really upset you because you I wanna be a good nurse you know I wanna show people that I wanna care for them and I don’t feel like I’m giving that to them because I haven’t got the time and I think that’s when you are really tested because you’re not thinking about your values you've not got enough time to give the kind of care that you want to give so that’s when you’re most tested. [*sic*] (P 13 Female, Band 5)

## Discussion

This is the first study to explore the relationship between value congruence, wellbeing and patient care and safety for RNs using in-depth, qualitative methods. The study found that there is alignment of personal and professional values of RNs. However, there were several areas of incongruence that RNs experienced between their values, and their supervisors and work environment, and between the organisation’s values and work environment. This incongruence was perceived to negatively impact upon the relationships between quality of patient care and safety, and RN wellbeing.

This study builds upon previous literature assessing RNs’ values by providing depth and understanding of the association between personal and professional values (Riklikiene et al., 2017). Personal and professional value alignment was reinforced: RNs believed there to be very little difference between their own personal values and professional values, and that having an inherent set of values was integral to the profession. This finding is supported by the literature reporting on RNs’ personal (Horton et al, 2007) and professional values (Weis and Schank, 1997). This alignment of the personal and professional values for nurses may lead to further ramifications for personal wellbeing as the sources of value incongruence at work cannot be separated from professional values.

Our findings contribute to the existing literature by describing the relationship between value congruence, wellbeing and patient care and safety. This adds to existing knowledge that value incongruence is linked with poorer staff wellbeing (Verplanken, 2004), and higher staff turnover (Gates and Mark, 2012) as it provides supporting accounts which show how these concepts are connected. Previous qualitative research established the relationships between burnout and patient care and safety for physicians as being potentially circular (Hall et al., 2016). This finding is supported by the current study, and together it suggests that value incongruence may be one catalyst for this negative cycle of high levels of burnout amongst nurses, and poorer perceptions of patient care and safety.

Further, the service pressures RNs described in this study which eroded values-based practice (i.e. staffing levels and external policy context) were also identified in a review which focused on contributory factors to patient safety incidents (Lawton et al., 2012). This finding supports the relevance of value congruence in the context of the work environment and patient safety implications. This study highlights the different contexts of pressures for nurses of different bands. Nurses in higher bands seemed more able to view service pressures within the wider context of external policies.

These findings raise important implications for supporting staff wellbeing within the current system. It is important to consider how to support nurses’ personal and professional values due to their close alignment: values-based recruitment and employment would support this endeavour. The incongruence identified by RNs between an organisation’s values and their work environment was considered to be related to poorer wellbeing and a poorer quality of care and safety for patients. Currently nurses are being recruited on the basis they hold the values of the organisation, through values-based recruitment (Department of Health, 2012), however, this study demonstrates if these values are not upheld within the work environment there will be a negative impact upon wellbeing and patient care and safety. This relationship is further supported by longitudinal qualitative research following students to newly qualified nurses: newly qualified nurses experienced burnout, disillusionment and planned to leave the profession because the work environment prevented them behaving in line with their values (Maben, Latter and Clark, 2007). Our findings suggest that organisations must support an environment that is aligned with the values of the nurses recruited. If they do not do this, they risk making nurses vulnerable to this potential negative cycle of poor wellbeing and burnout leading to poor patient care and safety (Hall et al., 2016) relating to value incongruence.

### Strengths and limitations

A strength of this study was the diverse sample across different specialities of RNs, a range of experience (i.e. bands and job titles), and geographically diverse Trusts. Despite this diversity the majority of RNs included within this study were White, female and Band 5: there was not the diversity in ethnicity and nationality that exists in the current nursing workforce. Although a telephone interview method was adopted with the aim of being as accessible as possible, further research should explore these findings with these harder to reach groups.

Future research should also explore the possibility of a values-based intervention to support nurses, as this study shows a relationship between value incongruence and wellbeing, patient care and safety. So, this may be an effective tool for supporting wellbeing, and improving patient care and safety.

## Conclusion

In summary, the current study has created a greater understanding of the relationship between perceived nurse wellbeing and patient care and safety, by demonstrating the influence of value incongruence upon these concepts for RNs. RNs reported that despite personal and professional value congruence, they often experienced incongruence between their own values and the values they perceived their supervisors to have, and a mismatch between working in line with their values within the work environment. Furthermore, organisational values were perceived to be challenging to uphold within the work environment, with several barriers described as preventing RNs’ ability to work in line with their values. This finding is important for the practice of values-based recruitment and the potential for values-focused interventions to support nurse wellbeing.

## Key points for policy, practice and/or research

- RNs’ personal and professional values were closely aligned.
- The values of hospital organisations were described as being incongruent with the RNs’ work environments.
- RNs experienced perceived value incongruence in different forms and described this as having an impact upon wellbeing and patient care and safety.
- As nurses are increasingly being employed through values-based recruitment, organisations need to ensure that the work environment and the organisations’ values are aligned to support RN wellbeing and patient care and safety
- Future research should explore the use of a values-based intervention to support wellbeing and patient care and safety.
